# An Embedded Wireless Sensor Network with Wireless Power Transmission Capability for the Structural Health Monitoring of Reinforced Concrete Structures

**DOI:** 10.3390/s17112566

**Published:** 2017-11-07

**Authors:** Luca Gallucci, Costantino Menna, Leopoldo Angrisani, Domenico Asprone, Rosario Schiano Lo Moriello, Francesco Bonavolontà, Francesco Fabbrocino

**Affiliations:** 1 TME s.r.l.—Test and Manufacturing Engineering, via C. A. Dalla Chiesa—81050, Portico di Caserta (CE), Italy; ing.lucagallucci@gmail.com; 2 Dipartimento di Strutture per l’Ingegneria e l’Architettura, University of Naples Federico II, Via Claudio 21, 80125 Naples, Italy; d.asprone@unina.it; 3 Dipartimento di Ingegneria Elettrica e delle Tecnologie dell’Informazione, University of Naples Federico II, Via Claudio 21, 80125 Naples, Italy; angrisan@unina.it (L.A.); francesco.bonavolonta@unina.it (F.B.); 4 Dipartimento di Ingegneria Industriale, University of Naples Federico II, Piazzale Tecchio 80, 80125 Naples, Italy; rschiano@unina.it; 5 Dipartimento di Ingegneria, Università Telematica Pegaso-Piazza Trieste e Trento 48, 80132 Naples, Italy; francesco.fabbrocino@unipegaso.it

**Keywords:** structural health monitoring, wireless sensor network, embedded sensors, wireless power transmission

## Abstract

Maintenance strategies based on structural health monitoring can provide effective support in the optimization of scheduled repair of existing structures, thus enabling their lifetime to be extended. With specific regard to reinforced concrete (RC) structures, the state of the art seems to still be lacking an efficient and cost-effective technique capable of monitoring material properties continuously over the lifetime of a structure. Current solutions can typically only measure the required mechanical variables in an indirect, but economic, manner, or directly, but expensively. Moreover, most of the proposed solutions can only be implemented by means of manual activation, making the monitoring very inefficient and then poorly supported. This paper proposes a structural health monitoring system based on a wireless sensor network (WSN) that enables the automatic monitoring of a complete structure. The network includes wireless distributed sensors embedded in the structure itself, and follows the monitoring-based maintenance (MBM) approach, with its ABCDE paradigm, namely: accuracy, benefit, compactness, durability, and easiness of operations. The system is structured in a node level and has a network architecture that enables all the node data to converge in a central unit. Human control is completely unnecessary until the periodic evaluation of the collected data. Several tests are conducted in order to characterize the system from a metrological point of view and assess its performance and effectiveness in real RC conditions.

## 1. Introduction

Since buildings and infrastructures are potentially subjected to environmental degradation or hazard-induced damages, it is necessary that safety, functionality and durability requirements be met throughout their service life. To guarantee efficiency and safety, an effective method consists in monitoring some structure-related properties that can be, in turn, associated with the local or global integrity of the structure itself. The range of possible measurements is wide and depends on the type of structure being investigated; as an example, health monitoring techniques can be based on measures of: local stresses or strain field; inclinometric measures; locations, amplitude and pattern of cracks; local rotations and displacements; temperature or corrosion of metal components. Based on the measured parameters, health monitoring of civil infrastructures can determine the location and severity of damage according to four levels of damage identification [1], as follows:

- Level 1: determination that damage is present in the structure
- Level 2: determination of the geometric location of the damage
- Level 3: quantification of the severity of the damage
- Level 4: prediction of the remaining service life of the structure.

In this context, a continuous real-time monitoring of such properties allows for a reliable and comprehensive knowledge of the integrity of in-service structures, entailing the implementation of optimal solutions for planned maintenance while minimizing downtime or damages due to hazardous events. Indeed, a significant portion of safety evaluations is currently based on periodic physical inspections, sometimes appearing inadequate or costly. The current practice mainly relies on Non-destructive Damage Evaluation (NDE) methods, because only a limited knowledge about structural properties of in-service structures is available in real time. In particular, nowadays, structural health monitoring (SHM) is implemented in reinforced concrete (RC) members through various techniques that are based on different operating principles. These have advantages and disadvantages depending on the specific environment where the structural component is located and the overall cost of the SHM technique itself.

Passive sensors have been widely studied in recent decades and mainly consist of metallic elements embedded in a given structure. Their resonance frequency changes through physical and geometrical variations that are associated with alterations in the mechanical properties of the structure itself [2]. Reliable solutions have also been introduced by employing mechanical-deformation-based techniques, which typically enable the analysis of the stress state in rock masses, metals, and plastics through the restoration of the deformation field around a predefined septum created by the user. Recently, the use of strain gauge sensors has been implemented to analyze and monitor the mechanical deformation of structures subjected to static and dynamic loads. Strain gauge sensors guarantee very high accuracy in terms of the measurement of strain which is calculated from the electrical resistance variation associated with the deformation of the support onto which they are bonded.

Further evolution has been achieved using piezoceramic sensors. These are applied externally to the structures being tested and are then correlated with the deformations of the outer surface to which they are attached. Piezoceramic sensors represent one of the first attempts to utilize embedded sensors in structures. They are typically used for vibrational analyses that exploit the piezoceramic effect. This effect allows the vibrations to be converted into an electric signal which, with appropriate filtering, tracks the acoustic events generated by the formation of internal cracks. More advanced techniques involve the use of two piezoceramic transducers, with the first acting as an acoustic emitter (by means of suitable electrical signals) and the second as a receiver. By analyzing the received acoustic waves transmitted from the first transducer through the structure, it is possible to conduct an analysis of the variation of the structural properties (from the density up to internal failures) that are related to the distortion of the waves propagated in the medium during the transmission [3].

The use of accelerometers is nowadays referred to as a continuous acoustic analysis, with concrete structures (pre-stressed and reinforced by cables) increasingly popular thanks to the growing interest in MEMS devices [4]. Distributed in key locations along a structure, each sensor is cabled to an acquisition unit, which continuously monitors the system without collecting data until there is a trigger event, e.g., an acoustic wave exceeds a predefined threshold. These sensors are effectively used to quantify the energy released by failure events that affect the overall dynamic behavior of the structure.

Over the past two decades, a large number of applications have made use of fiber optic sensors to monitor both new and existing large infrastructures. With a particular focus on pre-stressed structures, fiber optic sensors allow mechanical stresses to be analyzed during the construction phase (including all the deformations caused by physical shrinkage) and throughout the service life of a structure by monitoring its response to variations in applied loads. One example is the Tsing Ma Bridge where, in 2003, 40 fiber Bragg gratings (FBG) were installed on the hanger cable, rocker bearing, and truss girders to monitor the dynamic strain and temperature [5]. The *surveillance d’ouvrages par fibres optiques* (SOFO) system, literally “structural monitoring via optical fibers”, is a widely-used approach that employs optical fibers to conduct an analysis of mechanical deformations through the differential length between two fibers, one embedded in the structure and another that is free to move [6].

More recently, effective solutions for measuring structural properties and/or induced damage in RC structures, as proposed by Murthy [7], have made use of battery-less, embedded sensors which, for instance, make a direct analysis of possible steel rebar corrosion. As the sensors are wireless, manufacturing and installation costs are kept low. Furthermore, the absence of wiring makes the system totally maintenance-free and, at the same time, is a safer solution, since the embedding of the sensors is not an issue in terms of the stiffness of the structure [8,9]. However, this solution is unable to provide continuous monitoring of a structure, because the sensors can only be interrogated manually. The work by Ceriotti et al. [10] reports on the implementation of a wireless sensor network (WSN) for monitoring structural parameters. The network was comprised of different interconnected nodes, with each one capable of providing different measurements, e.g., temperature. This solution appeared to be very innovative, being a first step in the path to continuous check-ups, although it lacked the capacity of embedded measurements.

Even though the considered approaches face the problem of having a system for the continuous and embedded monitoring of structures over long time periods, they do not provide a comprehensive solution that solves all the related issues. In particular, Murthy’s study presents an interesting approach, but it does not allow continuous monitoring over time; on the contrary, Ceriotti’s approach solves this particular issue but does not permit the direct measurement of the phenomenon under analysis. As a consequence, the definition and implementation of such system is still an open challenge and research efforts are required to overcome typical issues such as effective embedment and continuous monitoring of the RC structures over time. In this context, the present paper intends to evolve the ideas presented in the previous solutions, while limiting some of the disadvantages, thus achieving a system that try to solve the MBM paradigm efficiently.

## 2. Problem Statement

Despite a wide variety of SHM solutions, a cost-effective structural monitoring technique that allows for the direct and continuous measurement of the stresses or other structural parameters within a RC structure throughout its service life is still required. Indeed, many of the solutions mentioned above are characterized by some limiting factors and drawbacks. For instance, low-accuracy and the reduced lifetime of passive sensors have been acknowledged in several studies [11], while stress-based methods typically need physical intervention in the structure, which requires a long execution time. Moreover, these SH solutions are unsuitable for either continuous monitoring or critical installations such as dams, power plants, or other structures at biochemical risk. Despite their accuracy, strain-gauge measurements are typically characterized by issues with regard to their installation, conservation over time, and the limitation of the measurements that are only possible for a portion of the structure that is generally referred to an external surface [12]. On the other hand, piezoceramic sensors represent a significant improvement in this field, but they only perform an indirect measurement of the accumulated stresses, which is carried out through a signal scattering analysis [13]. In the case of accelerometers, drawbacks are related to signal filtering operations required to remove superimposed noise and all other uncorrelated events [14].

In addition, all the methods discussed above are indirect; only the use of optical fibers allows the direct measurement of the deformation of a structure (and so the internal stresses), but with high costs in terms of both the installation and the measurement equipment [15].

It appears that an effective SHM solution should: (*i*) provide direct measurements of the variations of the mechanical properties in RC structures; (*ii*) implement economic sensor systems that would achieve the MBM in a flexible and effective manner; and (*iii*) allow the deployment of a large number of sensors to guarantee the global structural monitoring in several locations of the structure and track and analyze their evolution over time. Sometimes this issue is addressed as “A to E” paradigm, which is something the current solutions are still a long way from doing.

## 3. Proposed SHM System 

The system proposed in this paper—an embedded WSN for continuous structural monitoring—combines some of the features of the previous implementations and aims to overcome their weak points. A WSN is presented here that is capable of directly measuring concrete properties thanks to the embedding of sensors in the node units of the structure; with their wireless capability, the nodes can be safely integrated in a building without causing any safety issue. The wireless configuration is also extended to the node recharging, thanks to an inductive wireless power transfer module. Furthermore, the analysis is performed directly on the desired parameters, tracking all their changes over a long period of time thanks to the ultra-low power configuration. 

Once integrated into the structure, each node provides local information (e.g., stress values, temperatures etc.) while at the same time all nodes, thanks to the interconnected nature of the system and a data processing stage, give the “health” status of the whole structure. This feature is facilitated by the absence of connection cables and allows for an autonomous and continuous control of the structures. Specific devices used to track dynamic events, e.g., accelerometers, are not considered in this work due to their triggering and data recording characteristics. In particular, in the proposed monitoring system, data acquisition frequency is set according to the in-service time of the structure (e.g., weekly, monthly for stress/temperature measurements). On the contrary, in the case of dynamic properties evaluation, the acquisition periods would be no longer based on defined time intervals but rather on random events whose acquisition would start from specific “triggers”, such as acceleration thresholds.

The proposed monitoring strategy overcomes some of the limitation of current practice where physical inspections are conducted in different points of the building only after the damage occurs; in contrast, the interconnected network presented here can predict global consequences on the structure based on real time measurements before achieving a damaged state. Moreover, preventive actions are made possible by the fact that multiple measures are available in real time. In this way, regular and time effective maintenance strategies could be scheduled, resulting in effective implications for security and economy, and therefore for sustainability. A schematic representation of the whole SHM solution is shown in Figure 1.

The main element of the SHM is the node, i.e., a compact electronic system installed in the RC structure under test which, as generic application, consists of a reinforced concrete (RC) slab or column. The node is mandated to carry out the required measurements (e.g., stress and temperature) thanks to its installed sensors; obtained results are transmitted directly to the hub (referred to as *concentrator*). It’s worth noting that only stress and temperature measurements are considered in this study, but other properties might be measured through other devices properly equipped in the nodes. Moreover, the assembly described hereafter has been conceived for its implementation in a pilot small scale structural component made of concrete material. However, the outcomes of the tests are obtained under specific boundary conditions reproducing common in-service scenarios of whole RC structures.

The node includes four units (Figure 2): the *control unit*, the *transceiver*, the *inductive charging system*, and the *sensors*. The sensors, consisting of a load cell (LC) with a full scale range equal to 1 ton (≅10 kN) (type 3141-1T, supplied by Phidgets Inc.) and an analog temperature sensor (type MCP9700, supplied by Microchip Technology Inc.), are intended, respectively, to directly measure inner stresses and temperature of the concrete’s mass in which they are installed. The transceiver for communicating with the concentrator is a ME70-169 (supplied by Telit Wireless Solutions). The inductive charging system, which is used for recharging the on-board battery mandated to supply the node, is a CR18650 lithium-polymer (LiPo) battery (produced by Panasonic). Finally, the microcontroller unit (MCU), consisting of a Microchip microcontroller series Pic24, is a low-power DSP processor that not only acts as a controller for all the modules, but also carries out the measurements and transmits the related results to the concentrator, with an ad-hoc protocol written for this purpose.

The hub is the natural counterpart of the nodes. It is made up of a *PC station* and a transceiver, and the concentrator of the entire architecture is arranged in a topographically barycentric position (with respect to the nodes’ deployment), taking into account the transceiver coverage. As for the nodes, the transceiver is a Telit ME-70 and is connected to the PC with a USB adapter board (board included in the Telit Democase kit). The PC is equipped with a LabVIEW application that not only allows the automation of the system to be supervised via a selective wake-up/measure/sleep node sequence, but also the management of all the handshaking routines implemented to avoid collisions between more nodes transmitting simultaneously.

The architecture has a star network topology and provides the opportunity of covering not only the whole structure (*internal nodes*) from the basement to the roof, but also a small area covering multiple buildings (*external nodes*), thanks to the suitable communication range of transceiver modules. Transmission power, low operating frequencies, low bit rate, and intelligent management of the serial communication with an ad-hoc protocol allows a virtually indefinite number of nodes to be placed in a range of some hundreds of meters. The system is fully automatic, and the required interactions with the final user are limited to two situations:

1. on the hub, during the analysis of the reports (Figure 3);
2. on the node, during the recharging procedures.

Thanks to the PC application, the user can analyse all the data collected from the hub, both in graphic and chart form, for both monitored quantities. A data plot on a time base can be made to analyse the evolution of the temperature and stress measurements, from seconds up to years, in order to quickly identify faults and their causes. Information from the nodes can also be plotted, such as the battery voltage, with all the stored data indexed by the relative node. With the same application, the nodes can be interrogated individually once they are woken up and some configuration parameters can be set, e.g., the idle/sleep time intervals. In any case, operator participation is all but unnecessary: the hub is self-sufficient and operates the network automation. Moreover, a database of collected data is retained and its exportation is possible, thus allowing it to be processed at a later stage and/or with third-party software. 

The second interaction between the network and the operator occurs during the maintenance procedures of the nodes, which consist of a recharging session of the on-board battery and take place in proximity to the node itself. The nodes, at time intervals of about 10–12 months (depending on the measurements frequency), run out the charge accumulated in the battery and need to be recharged. The recharging session is performed with an induction charging system installed for variable time intervals (two-six hrs in the proposed solution) on the outer surface of the slab in which the node is installed. The same procedure can be used to wake up a node, which, for the majority of time, is kept in a deep sleep state to preserve energy and is therefore not connectable.

To complete the description of the system, some details about the main improvements achieved are presented below. 

### 3.1. Inductive WPT Charging System

When defining the power source for the nodes, battery-less solutions have been discarded, as they would prevent a WSN-architecture with adequate coverage; the choice for battery powered solution made the design of the recharging system a key factor. Moreover, the embedded implementation of the nodes has highlighted the need for a wireless charging system. RF power harvesting systems were discarded, since the low efficiencies evidenced in the state of the art became even worse due to the filtering action of the concrete in the presence of high frequency signals. Approaches based on thermoelectric effect, solar power, vibrations, and harvesting proved also unfit, because of very low temperature gradients, need for cables, and insufficient energy provided by the low efficiency, respectively.

A wireless, high-efficiency, low-frequency charging system is therefore required to transfer the energy required by the nodes. All the literature about wireless power transfer (WPT) methods, in particular the work by Angrisani et al. [16,17,18,19,20], proposes an inductive WPT method based on very low frequency signals to transfer energy within a range up to 30 cm, suitably outperforming commercial inductive chargers, whose operating range is limited to 5 cm.

The system proposed in Figure 4 is a push-pull inverter in the primary circuit and a full bridge rectifier in the secondary circuit. The transmitter circuit (primary) core is an inverter based on the principle of a relaxed oscillator. In particular, the MOSFET works in a push-pull configuration, turning on and off the two branches of the circuit upon the polarity of the signal in the capacitor that is installed parallel to the coil. Voltages and currents in the resonant inductive/capacitive circuit are characterized by a natural resonant frequency which depends on the capacity of the cell and mutual inductances of the system. This frequency has been set in the design stage to 100 kHz by suitably setting the values of inductance and capacitance.

The transmitting device is a coil comprised of four turns made with a 2.5 mm^2^ stranded cable wound up to a 14-cm diameter. This is connected to the two branches of the inverter, while the central tap is connected to the power line.

The main advantage associated with relaxed oscillators is the automatic tuning capability, which allows the automatic following of the maximum power point (MPP), thus guaranteeing the transmission of maximum power. The choice of a fixed frequency DC/AC inverter, without a feedback auxiliary circuit for the frequency control, would lead to power losses. These are caused by inevitable detuning effects (involving a frequency mismatch) linked to: (i) parametric variations of the components; (ii) intrinsic tolerances; and (iii) geometrical variations (in both the system itself and the mutual position of the primary and secondary circuits).

The *receiver circuit* is made symmetrical to the transmitting circuit (in order to have the same resonant frequency). It includes an identical coil (four turns, 2.5 mmq, and 14 cm) and the same capacitor pack. A full-bridge rectifier made with high-frequency diodes completes the proposed circuit, thus providing a very compact form, which is important for the successive embedment.

### 3.2. Low Power Architecture 

In order to guarantee a battery life that is long enough to avoid the need for frequent recharging sessions, an adequate power design (PCB level) has been developed by working on the power consumption of the components first and, then, on the selective enabling. A great effort has been made in this second stage [21]. From a high-level point of view, the circuitry involved in the supply management can be divided into the following main blocks:

- *wireless recharging system*, including receiving coil and conditioning circuit;
- *accumulator circuit*, composed of a three-stage system made up of input voltage regulator, battery charge regulator, and LiPo accumulator;
- *device voltage regulating circuit* composed of the MCU voltage regulator (very low quiescent current to enable ultra-low power standby) and a peripheral voltage regulator (to disable unused external peripherals during the sleep condition).

Joining this low-power circuitry with the deep sleep MCU capability and the Li-Po battery assures very low discharge rates thus allowing very long lifespan to be achieved. The proposed system in the deep-sleep condition achieves measured consumptions lower than 1 µA; in particular, average current values as low as 415 nA have been experienced, if compared to the 150 mA peak values measured during the working state). When considering a realistic scenario with a measurement rate of one measure per week (corresponding to a duty cycle of 0.001% if 5 s average operating time is considered), the whole power consumption is equal to 2.437 mWh/week. This value represents only 1% of the battery self-discharge rate (8–10% capacity/month, i.e., 277.5 mWh/week). In a long-term monitoring configuration, the expected operating cycle can be calculated by making an acceptable approximation in considering the electric consumptions as zero and concerning only the battery shelf life [22,23].

### 3.3. MCU Firmware and Communication Protocol

The microcontroller unit has two different working states, *sleep* and *wake-up*. In the *sleep* mode, the unit is put in a low consumption state where both external and internal peripherals are electrically disabled (cutting off their supplies), but the real-time clock calendar (RTCC) which stores the wake-up date, and the watchdog timer, which wakes up the unit if a programmed timeout has elapsed (to prevent a misconfiguration that leads to infinite sleep sessions). As stated above, this status is needed to allow a very long battery life and reduce the number of charging sessions [24,25].

When the preset date is reached, the unit exits from the sleep condition and enters the wake-up state. In this condition, all the peripherals are turned on and a three-phase handshake/measurement/data transmission session occurs. The first phase is a handshake session where the unit introduces itself in the network, sending a request signal to the concentrator. The message is then repeated (with a random time delay to avoid conflict with other nodes) for a finite number of iterations until an acknowledgement message is received from the concentrator, which contains the information on the number of measurements to carry out and their accuracy. The RTCC is then updated with the current time stamp and the wake-up date. Once the message is received from the node, it carries out the measurement routine, conducting multiple acquisitions to reduce acquisition noise, and then goes back into the sleep mode. If other nodes are asking to log in to the network, each of which having a univocal ID that enables the concentrator to differentiate the requests, the concentrator registers them in a queue and processes their requests with a time priority criterion.

An additional condition can take place, even if it does not belong to the regular activity condition, and is represented by a battery recharge session. Once the inductive charging system is turned on and installed in proximity to the node, the start of a recharge leads to a forced node wake-up, which automatically sends a message on the state of the battery charge and performs a quick measurement session. This information is then processed from the concentrator, which notifies the user about the end of the recharging session (and the possibility of removing the charging system).

### 3.4. Load Cell Sensor and Low Powering Considerations

Load cell sensors are widely used in force measurements, and are de-facto utilized most when measuring weights. Their use in the SHM field is relatively new, and this paper moves this idea forward, proposing their utilization in an embedded configuration with a very simple load cell type as a first attempt of this application.

The use of LCs is preferred when dealing with large forces, thanks to the wide range of scales available nowadays, even in commercial sensors. There is an increased interest on these sensors, since the cost of even a high range unit, make them an interesting solution for inner stress measurement of concrete masses. In this study, a 1000 kg range load cell sensor has been employed. The high required power supply is the main problem associated with the integration of LCs in the considered nodes; this way, a preliminary performance assessment in the presence of low power supply is mandatory.

As shown in detail in the successive Section, investigations on the low powering of the proposed LC (a Phidget 3141-1T) have been conducted by powering the sensor from a 3 V supply. The lower voltage, compared with a 5–18 V minimum supply and a 9–12 V recommended supply, produced only a limited reduction of the operating range. Worse uncertainty and noise were not observed.

Further studies were carried out to enable the direct embedding of the sensor in the concrete mass. A metal casing was designed to improve the sensor protective structure in order to avoid unwanted compression on the sensor’s sensitive surfaces, which would have led to misreading. As shown in Figure 5, the cover consisted of two plates, the first one on the bottom to improve the structural strength of the bottom lid, and the second one, which is a cap to distribute the force impressed on the top face, only on the sensor’s button (the sensitive area) and not the upper face.

## 4. Metrological Characterization and Performance Assessment

Different experimental tests were conducted to metrologically characterize the LC sensor and assess the performance and effectiveness of the inductive charging system. Three sets of tests were conducted: one set on the LC in free air; two sets on the inductive charging system, one in free air and the other in a concrete environment.

### 4.1. Free Air Tests 

#### 4.1.1. LC Characterization

LC characterization included two tests, the first of which aimed at assessing the LC effectiveness under reference loads applied in off-specification, low-power configuration. As previously stated, major modifications were made to simplify the circuit, including power consumption improvements. A first change was the reduction of the LC voltage supply. This helped to avoid the insertion of inefficient DC/AC circuits to step up the inadequate battery voltage (3.7 V, unsuitable for the 12–18 V LC required supply). Consequently, a more efficient system was achieved, reducing the LC power consumption. A non-working hypothesis was immediately discarded, since LCs are based on Wheatstone bridges, involving resistive sensing elements. On the contrary, reduced values both of sensitivity (due to saturation effects close to the input range values) and output voltages (because of the ratiometric nature of the LC sensor) were expected in the following tests.

A second test focused on the eventual artefacts due to the structural redesign of the sensor, i.e., the metal casing added to the LC. In particular, the effects of redistribution of the acting forces and the elastic behaviour of the aluminium casing have been assessed.

The test was carried out by means of universal servo-hydraulic machine with 500 kN maximum force capability (Figure 6). The instrumentation included an E3648A PSU (produced by Agilent, Santa Clara, CA, USA), an Agilent 34401 DMM, and a breakout board of the same instrument amplifier used on the node, namely an INA826 with a gain of 500 to maximize the output swing.

Four different experimental configurations have been considered, including: (i) a reference test with an uncased LC and standard supply; (ii) an uncased low-powered LC; (iii) a cased LC standard supply; and (iv) a low-powered cased LC. Data were acquired in three steps: a continuous sweep 0–10 kN load (elastic response of the DUT); an ascending 20 steps 0–10.5 kN load; and a descending 20 steps 0–10.5 kN load (alternate step directions are used to highlight eventual hysteresis effects).

Figure 7 shows the evolution of the differences, expressed in relative percentage terms, between the LC voltage nominal value (calculated as theoretical value from the sensor specifications at the corresponding output) and the measured one ∆V_LC_ = (V_NOMINAL_ − V_MEASURED_)/V_NOMINAL_ versus the applied force. In particular, four curves are shown: “*10 V Ori*” refers to the bare LC sensor operated at 10 V Supply, “*10 V Mod*” is the cased sensor @ 10 V supply; similar considerations hold with “*3 V Ori*” and “*3 V Mod*” for tests involving 3 V supply.

With regard to mechanical properties, the tests confirmed the negligible effect of the aluminum case (top and bottom cover), as highlighted by the remarkable concurrence of the 10 V and 3 V “Ori” and “Mod” curves in Figure 7. Considered results were achieved as average value of 30 repeated measurements for each load condition; to better appreciate the agreement among the different configurations, an inset showing the magnitude of error bar (equal to 1 experimental standard deviation) associated with each measure is given in Figure 7.

Differently from what expected, tests revealed that reducing the power supply does not affect the accuracy of the LC only in a specific range of applied force. A reduction of the sensor’s output range has indeed been experienced from 1000 kg to 460 kg; in particular, the nominal full-scale range (1000 kg @ 12 ÷ 18 V) is reduced, respectively, to 815 kg (8 kN) @ 10 V and 460 kg (4.5 kN) @ 3 V (Figure 7). Anyway, this effect can be easily compensated by choosing an LC with a greater range. An error on the lowest part of the scale is also noticed because of the superimposed noise which became comparable with the useful signal, in low output conditions.

Free air tests aimed also to determine the calibration curve of the LC sensor in a low-power configuration by means of a polynomial interpolation, which has been then transferred and saved in the MCU. By using the Matlab curve fitting tool, the calibration curve has been split into three regions: (*i*) a region I belonging to the 0–4.5 kN range, represented with an 8th order polynomial curve; (*ii*) a region II which is a linear knee region in the range 4.5–5 kN used to join the region I and III; (*iii*) a region III with an exponential function, in the range 4.5–10 kN (the saturation region, beyond the limit of the reduced range).

In Figure 8 the three regions of the calibration curve are shown; the black circles indicate the measurements from the characterization stage while the red curve is the polynomial curve representative of the region I, the blue one is the exponential curve of the region III and the light-blue curve represents the linear knee of region II.

The goodness of the calibration curve fitting (based on both a Matlab evaluation of the results and a conclusive cross-check of the calibration curve upon the input data) has shown very good results with a maximum deviation expressed in relative percentage terms equal to 0.5% in the region I, which represents the proposed working range, and a maximum of 3.5% in the region III. 

#### 4.1.2. Inductive WPT Charging System Characterization

The inductive charging system has previously been developed in the work by Angrisani et al. [16,17,18,19,20] and largely tested in free air; these tests have been reproduced as a reference for the subsequent tests in concrete.

**Figure 9:**
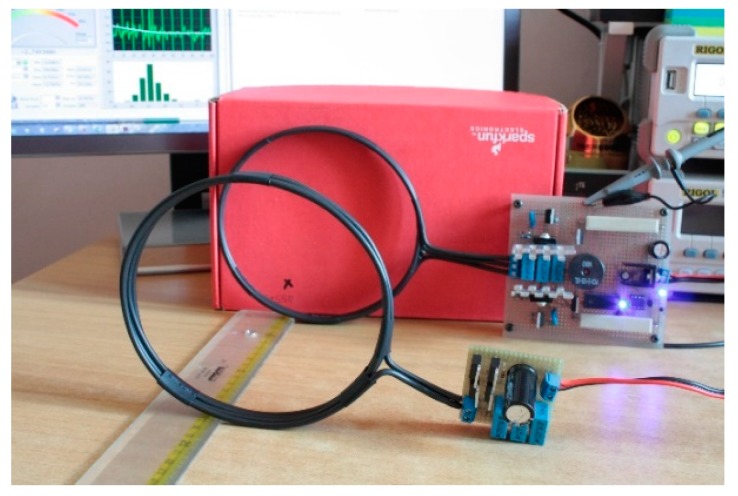
Inductive charging system test, free air.

**Figure 10:**
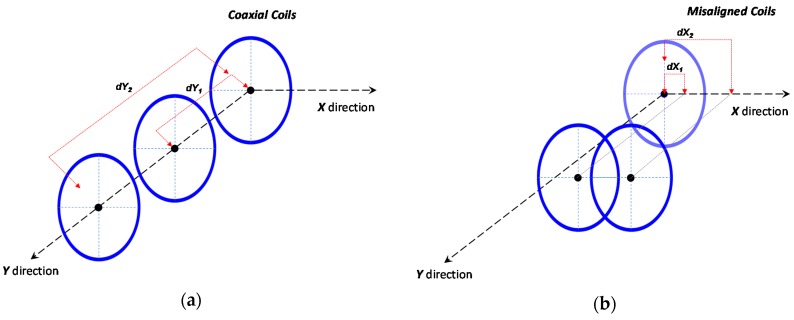
Cell displacement test, coaxial (**a**) and misaligned (**b**) configurations.

The test set-up showed in Figure 9 includes a DM3058E^TM^ digital multimeter (produced by Rigol, Beaverton, OR, USA) a Rigol DG1022U^TM^ frequency meter, and a PS-305D^TM^ DC power supply (supplied by Long Wei, Dongguan, China). The following measurements have been obtained and were aimed to consider real situation of charging operations in RC structures considering also different supply situations:

○ *Primary electrical absorption and free running frequency:* this test has verified that 110 kHz is a project constraint (measured 110.4 kHz ± 0.1% in all the range), total consumption of 263.1–674.6 mA (load off) and a 6 V minimum power supply were also measured.
○ *Secondary open circuit (OC) voltage (coaxial coils):* the secondary V_OC_ has been tested by loading the primary with the secondary circuit and varying both the distance (0–35 cm) between the coils and the primary power supply (6, 12, 15 V). The interval of interest of coil distance is approximately 10–15 cm (y direction, Figure 10a), representing a maximum plausible distance between an embedded node and an external charging system. Within this distance range, a minimum of 7 V is achieved by a 6 V supply @ 15 cm and a maximum of 31 V by a 15 V supply @ 10 cm.
○ *Secondary OC voltage (misaligned coils)*: with a primary power supply set to 12 V, the secondary V_OC_ is analyzed in this case by displacing the coils in the transverse direction of coil axis (*x* direction in Figure 10b) to simulate a recharging operation where (once embedded) a perfect alignment is unlikely to be achieved. The test is conducted considering different axial distances between coaxial coil centers (in y direction Figure 10a up to 35 cm) and different coil alignments in x direction (Figure 10b), keeping axis parallel. In particular, coil center alignments of 100–50–25–0% have been considered where the alignment percentage is calculated as (coil diameter − *X* distance between coil centers)/coil diameter; the value of 100% corresponds to the coaxial case while 0% is the full misalignment. In the 10–15 cm range of axial distance (*y* direction), we obtained an average 20% power loss @ a 75% alignment, 40% @ a 50% coils displacement, and a 76% loss @ a 0% alignment, thus confirming the goodness of the system if there is an inevitable misalignment during charging operations.
○ *Secondary short circuit (SC) current:* the output capacity has been tested by supplying the primary section at 12 V and shorting the secondary circuit; current values as high as 125 mA is reached at 15 cm, 365 mA @ 10 cm, and 678 mA @ 7.5 cm, respectively, thus matching the specifications of the required minimum node supply current (defined as 100 mA in the previous project constraints).
○ *Secondary SC current (on voltage regulator):* with the same primary power supply, the output capacity of the recharging system is tested here, shorting the output of the onboard node regulator: 321 mA @ 10 cm and 128 mA @ 15 cm.
○ *Battery recharging test, node recharging test:* final tests have been performed to recreate a recharging session of the node, isolating the controller unit first and then, in a further test, testing the whole node system. At a 12 V primary supply, 333 mA @ 10 cm and 131 mA @ 15 cm are achieved.

**Figure 11:**
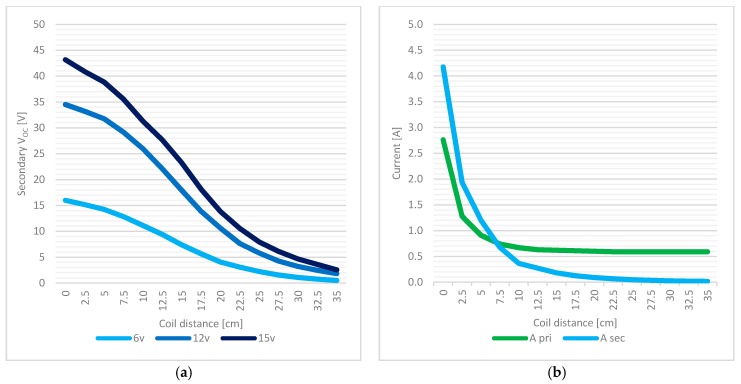
Inductive charging system characterization, free air and coaxial coils (coil distance is in *Y* direction of Figure 10a): (**a**) the secondary V_OC_ at different primary supplies, (**b**) the I_SC_ compared to primary I_SUPPLY_.

In Figure 11a the evolution of the secondary output voltage versus different coils distances is plotted for three different primary supply voltage values (6–12–15 V). In Figure 11b a comparison between secondary I_SC_ (“*A sec*”) and primary input current (“*A pri*”) is shown in the case of 12 V primary supply, which proved to be the best candidate for the final setup.

**Figure 12:**
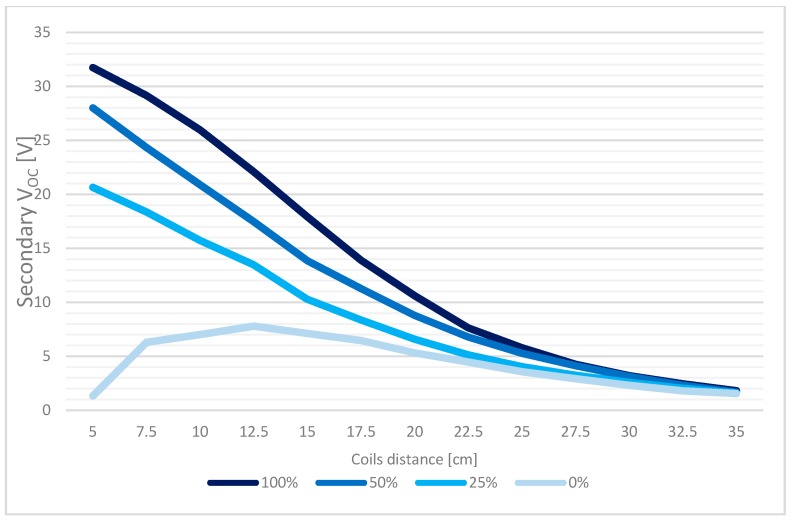
Inductive charging system characterization, free air and 12 V supply: secondary V_OC_ in the coil displacement test. Coil distance is in *Y* direction of Figure 10a; displacement is expressed in percentage values as the *X* direction of Figure 10b.

Figure 12 shows the sensitivity of the system to coils displacement and misalignment: the four curves at different axis alignment, expressed as percentage values as [(coil diameter−distance between axis centers)/coil diameter %], show the secondary output voltage by different coils distance when primary supply is equal to 12 V.

Tests outcomes first confirmed the matching of the built system with the one described in the reference papers [16,17,18,19,20]. Moreover, according to a possible scenario of a recharging operation at 10–15 cm distance, tests proved the effectiveness of the technique for recharging the node system even in case of an alignment percentage down to 25%, which represented the worst case.

### 4.2. Tests in a Concrete Environment

#### Inductive WPT Charging System Characterization

**Figure 13:**
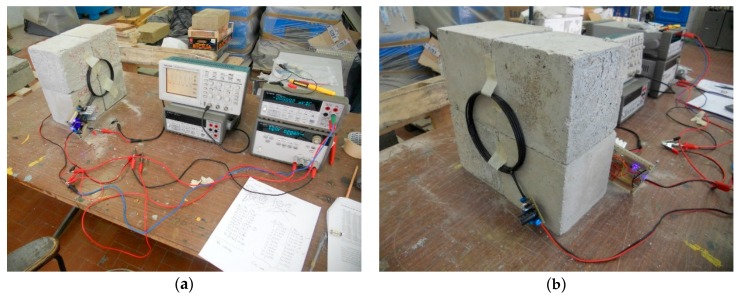
Inductive charging system test setup, concrete environment: (**a**) is the test bench set-up, including the required instrumentation; and (**b**) is a picture of the set-up of the coils.

Further tests have been carried out to prove the effectiveness of the inductive charging system in an actual concrete environment. A possible test set-up, which is useful for making multiple measurements with the same wireless unit, is represented in one (or more) concrete blocks that are large enough to avoid fringe effects on the sides of the coils of the inductive charging system (Figure 13). Multiple configurations have been implemented in the proposed set-up: (i) a set of four concrete blocks forming a wall of 30 × 30 × 15 cm (to simulate a concrete environment); and (ii) a 30 × 30 × 14 cm concrete block with an embedded 1 mm metal grid (to simulate a worst-case scenario of an RC environment); both the V_OC_ and I_SC_ have been measured by varying the misalignment (three step: 0–50–100%) and the power supply (10 step: 6–16 V).

At 15 cm @ 12 V_PRI_ (coaxial coils), a V_OC_ of 12.722 V is measured in the case of transmission in concrete which, compared to the air case where a 12.726 V is measured, represents a perfect match. Moreover, displacement behavior is analyzed in the same case of 15 cm @ 12 V, with displacements of 0%, 50%, and 100% implicating a reduction of the V_OC_ from 12.722 V to 10.188 V and 3.575 V, respectively. As stated before, higher voltages can be achieved by increasing the primary supply.

The I_SC_ has also been measured (even if an MPP test should be performed in a future set-up), confirming the results of the previous V_OC_ tests. There is a satisfying concurrence between the concrete and air set-up, with respectively 150 mA and 146 mA @ 15 cm/12 V/coaxial coils, and a variation in the misalignment in the three cases of 0–50–100%, respectively, of 146 mA, 100 mA and 32 mA.

**Figure 14:**
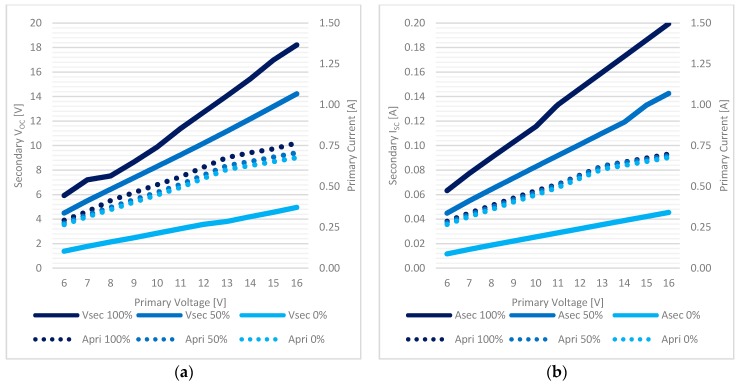
Inductive charging system characterization, concrete environment: (**a**) V_OC_, (**b**) I_SC_.

Figure 14 shows the evolution of secondary V_OC_(a) and I_SC_(b) versus primary voltage supply in three different alignment conditions (in *X* direction) where 100% is perfect alignment between coil centers and 0% is complete misalignment. Primary current is also shown in the same conditions. 

**Figure 15:**
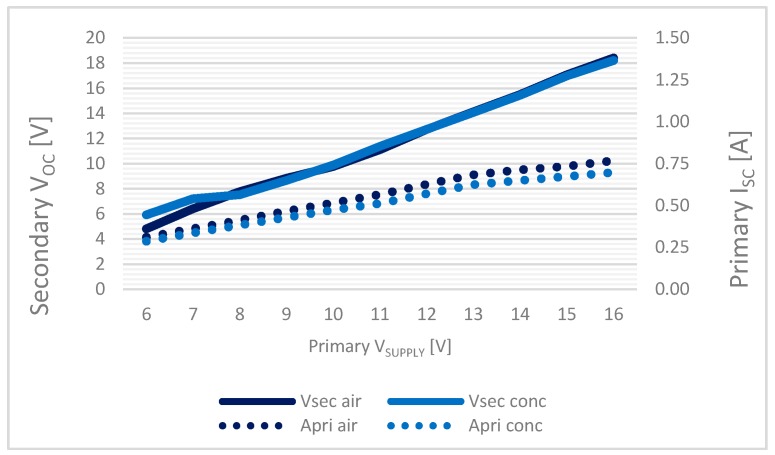
Inductive charging system characterization: secondary V_OC_ and I_SC_ in air and concrete environment.

A first goal has been achieved by analyzing the variation of the V_OC_ and the I_SC_ variation of the two test set-ups (air-concrete), where no differences are evidenced thus proving the complete transmissivity of the concrete at the used frequency range; as shown in Figure 15.

Even more interesting results have been achieved in the case of the transmission through a sandwich block of 4 cm concrete + 1 mm steel reinforcing grid + 9 cm concrete which represent a realistic scenario where concrete is usually reinforced with embedded steel grids and rebars, whose at first glance may act as disturbing elements for signal transmission. Normalizing the values by the coils distance (Y direction of Figure 10a), the results have shown a great improvement in the power transmission, which can be easily explained considering the model of the system as a transformer. Still referring to the case of coaxial coils and 12 V according to the normalized values, we have 0.848 V/cm in the case of air/concrete, and 1.388 V/cm in the case of reinforced concrete. This behavior can be explained by referring to the inductive charging system as an air transformer and not an antenna system. By increasing the permittivity of the medium, in this case embedding metallic elements as meshes or rebars, the power transmission is consequently boosted.

**Figure 16:**
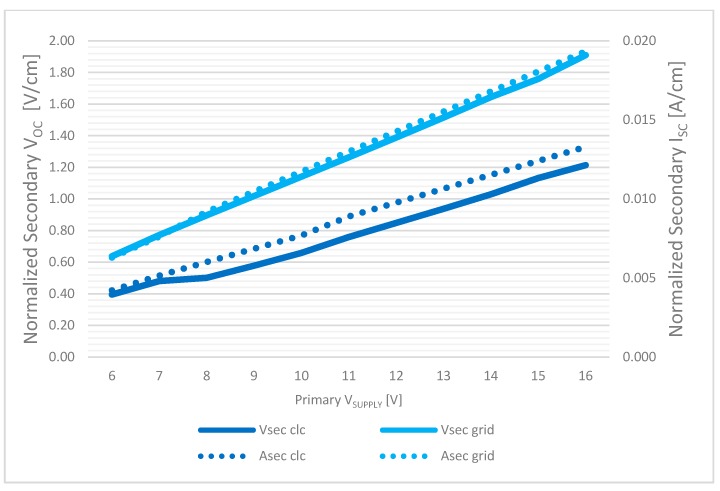
Inductive charging system characterization, normalized secondary V_OC_ and I_SC_ in concrete and reinforced concrete environment.

Figure 16 shows the secondary V_OC_ and I_SC_ normalized by the distance, as a function of different primary supply voltages in the two configurations above discussed: concrete slab (“*clc*”) and metal + concrete sandwich (“*grid*”).

This way, the proposed inductive charging system proved to be a valid candidate for wireless charging in an SHM implementation. Conducted tests, in fact, highlighted that nominal recharging performance was not affected by the installation in concrete environment. Moreover, the presence of steel elements as grid or rebars (typically involved in RC structures) results in outperforming the free air conditions, due to advantageous magnetic coupling with ferromagnetic materials. Finally, the influence of coils misalignment (*X* direction of Figure 10b), if limited to 50%, prove to be not significant, showing only a limited loss in performance that is almost irrelevant.

## 5. Combined Mechanical–Electrical Test in a Real Scenario

**Figure 17:**
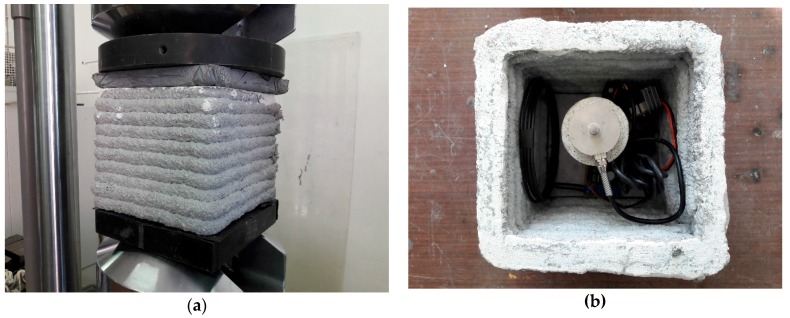
Concrete sensing element: (**a**) the device under test, (**b**) open element showing the enclosed system.

Final tests have been performed with the aim of assessing the performance and effectiveness of the proposed SHM system in a realistic scenario. Specifically, a 3D printed concrete casing has been produced. As shown in Figure 17, the casing has a dimension of 20 cm per side and is printed by nine layers of fast-setting concrete. The cube also has a removable top for further inspection. The system was then installed in the case, with consideration given to both the orientations of the antennas and the forces impressed on the block; for these reasons, a concrete pillar was installed to support the LC and let the forces distribute on the active sensing area.

The system has been tested in the same environments of Section 4 in order to compare the results within the operative conditions.

**Figure 18:**
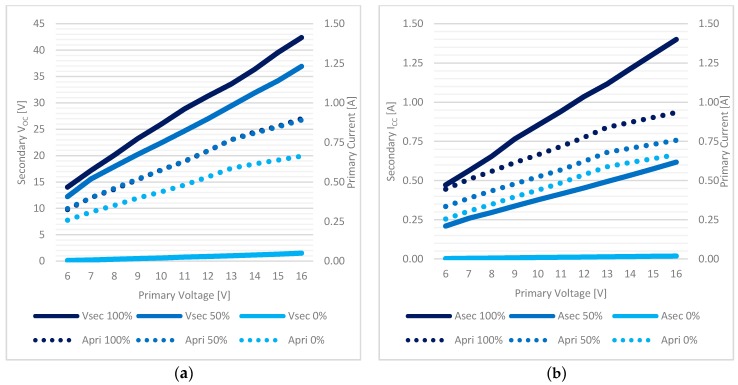
WPT analysis in the embedded system: (**a**) V_OC_ of the WPT system, (**b**) I_CC_.

The *inductive charging system* has been tested by measuring both the V_OC_ and the I_CC_ of the secondary WPT, with the primary current also analyzed. Tests have shown a perfect match with the previous results, confirming the lack of fringe effects in the previous tests, and a definitive confirmation of the transparency of the concrete to the 100 kHz frequency in the near field. A V_OC_ of 27 V @ 12 V supply and a 0.776 A I_SC_ were measured by the cube side. Displacement tests (50% and 0% of the radial distance) have confirmed the same amount of degradation of the parameters as in the previous tests. Figure 18 shows, in three different alignment conditions (100–50–0%), the measured secondary V_OC_ (a—”V*sec*”) and I_SC_ (b—”*Asec*”) as a function of different primary supply voltage, compared to primary current absorption (“*Apri*”).

The *LC sensor* has been tested in the same electrical working conditions of the developed node system: a 3 V supply with an amplifier gain of 500. The specimen has been tested with an applied force of 0–70 kN both in an ascending and descending ramp.

**Figure 19:**
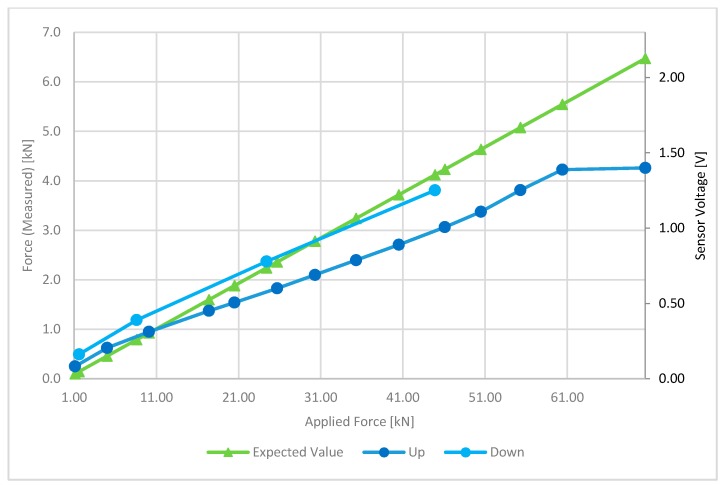
Load cell analysis of the embedded system during load and unloading phases.

Figure 19 reports the voltage measured from the embedded load cell (“*V*”) and the corresponding calculated force (“*Up*” and “*Down*”) values, as a function of a 0–70 kN ramp of applied force in the two different loading cases: ascending (“up”) and descending (“down”) ramp. The applied force is the force imposed by the testing machine onto the 20 × 20 cm^2^ area of the concrete element. By properly designing the applied load protocol (i.e., load steps and thresholds), it is possible to retrieve the force exerted onto the load cell area on the basis of mechanical considerations and areal ratio of the two components (concrete cover and effective load cell area). Therefore, the expected value is also plotted (green curve) and refers to the resistant force associated with the area of influence of the load cell sensor within the concrete specimen.

The mechanical test conducted on the embedded WSN system has shown a deviation of force readings limited to a range of ±20% with respect to the applied force after a 10 kN threshold. Loading and unloading steps have shown little difference, due to the occurrence of concrete irreversible deformations; a sensor saturation effect has also been noted, confirming the previous analysis in free air where a reduced range has been noticed after the reduction of the sensor’s power supply.

**Figure 20:**
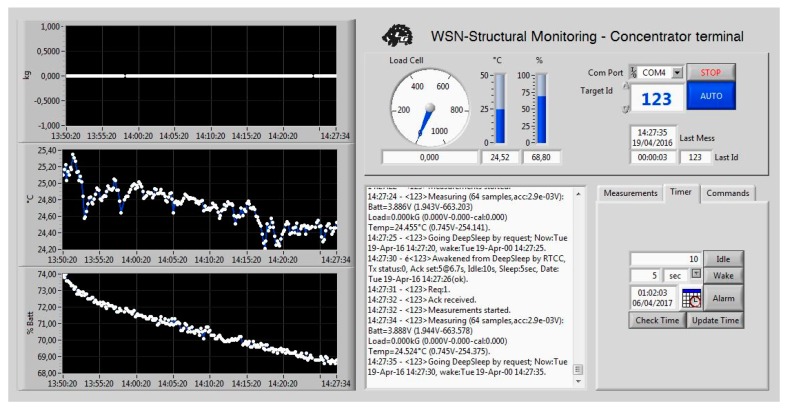
Short-term monitoring analysis.

A final test has been performed to analyze the transmissivity of the *wireless transmitter* which, as assumed, shows an irrelevant attenuation in concrete. A brief logging session with the developed software has been executed, confirming what has been assumed above (Figure 20).

## 6. Conclusions

In this paper, a structural health monitoring solution based on a wireless sensor network has been proposed aiming to enable the automatic and real-time monitoring of a whole RC structure. The network is comprised of wireless distributed sensors embedded in the structure by means of several nodes which can be placed in strategic points of the structure itself, such as slabs, beams or columns. 

The monitoring system is comprised of four main units: the control unit, the transceiver, the inductive charging system, and the sensors. In the present study, attention has been focused on a force sensor (load cell) and an analog temperature sensor; they are intended to directly measure the concrete’s inner stresses and the temperature of the mass in which they are installed, respectively. 

Several tests have been conducted on a small-scale sensing concrete elements. The tests concerned load cell sensor and inductive charging system characterization, both in free air and in concrete environment, trying to reproduce realistic in-service measurement and charging conditions. The free air test outcomes have revealed that, for the proposed inductive charging system, the involved values of voltages and currents are able to guarantee an effective wireless recharging up to 20 cm′15 cm of coil distance, even in case of an alignment percentage between coil centers of 25%, which represents a very a realistic scenario for in-service recharging operations. In terms of effects of concrete and steel material, it has been found that the proposed inductive charging system is not affected by the presence of a concrete medium, but is even more efficient with the presence of a steel element, being that a common situation in case the system is embedded in a RC structure. As final assessment, the mechanical test conducted on the embedded WSN system revealed maximum of ±20% deviation in the force readings compared to the applied force beyond a 10 kN threshold value. 

The results obtained represent a proof of the concept that should be validated also at the full-scale of representative RC structures. In particular, other fundamental aspects need to be addressed for the scale up of the SHM system which are currently under investigation by the authors, such as: sensor miniaturization, structural testing design, RC sample design, data validation over time, mechanical interactions between sensors and concrete medium, dynamic properties assessment, and battery effectiveness. 

As a future perspective of development, the SHM system presented in this study represents a first attempt to create an effective interconnected monitoring network based on a large-scale deployment of the sensors. The real-time analysis of all measurements provided by those sensors has the potential to make possible a comprehensive assessment of the health of the structure. Thanks to continuous monitoring, the system would also be able to conduct real-time failure signalling, which was previously impossible to achieve with a periodic pre-programmed and time-scheduled analysis/inspections. An even greater benefit that follows on from this implementation is that automatized monitoring can make early warnings possible thanks to the continuous evaluation of the variations along the time of the parameters that can lead to future failures. In this way, the proposed system contributes to the creation of smart structures according to the requirements of Industry 4.0. 

## Figures and Tables

**Figure 1:**
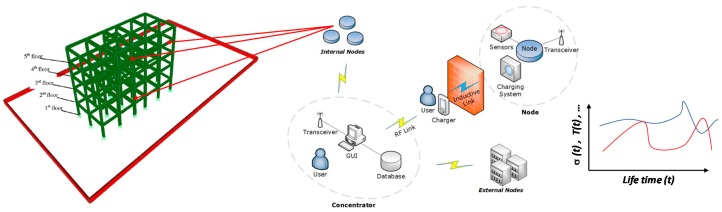
The WSN architecture.

**Figure 2:**
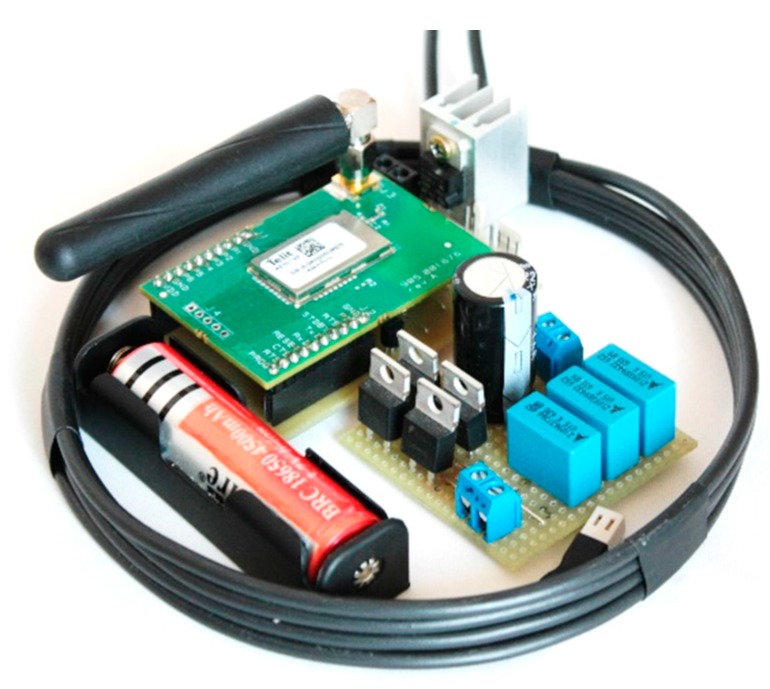
The node (uncased).

**Figure 3:**
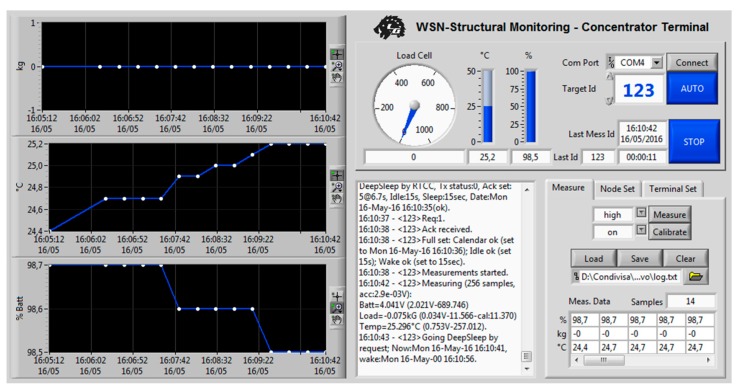
The hub terminal.

**Figure 4:**
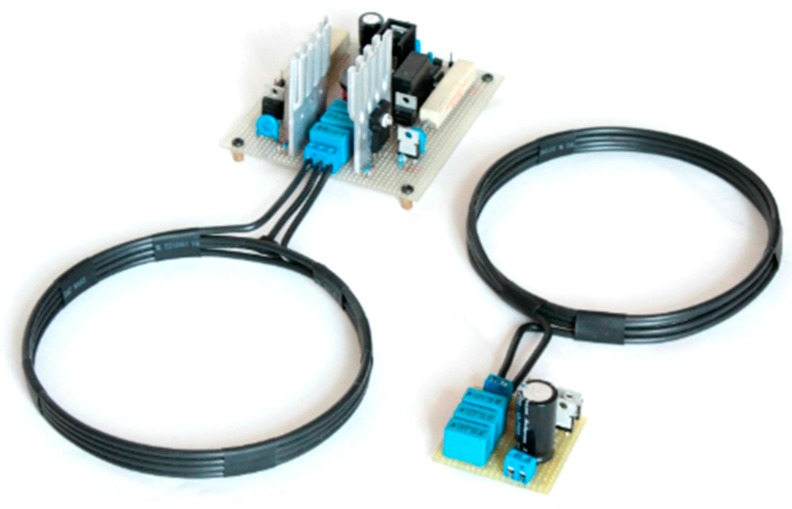
Inductive WPT charging system.

**Figure 5:**
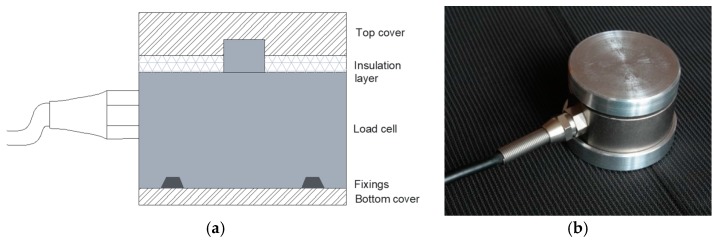
Load cell sensor, cased: (**a**) schematic representation, (**b**) final assembly.

**Figure 6:**
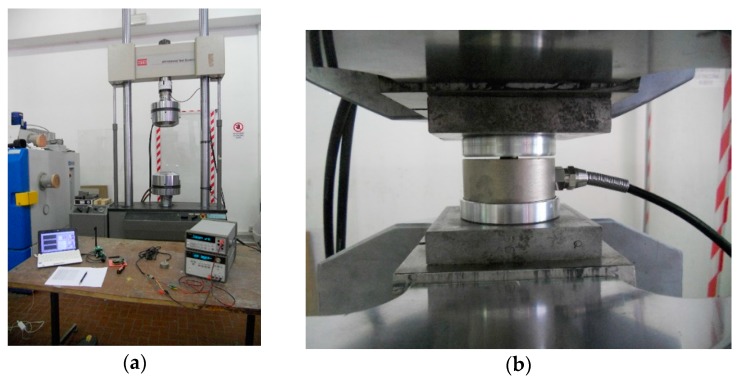
Load cell test set-up, free air: (**a**) experimental setup, including the MTS hydraulic press, and (**b**) detail of the LC under test.

**Figure 7:**
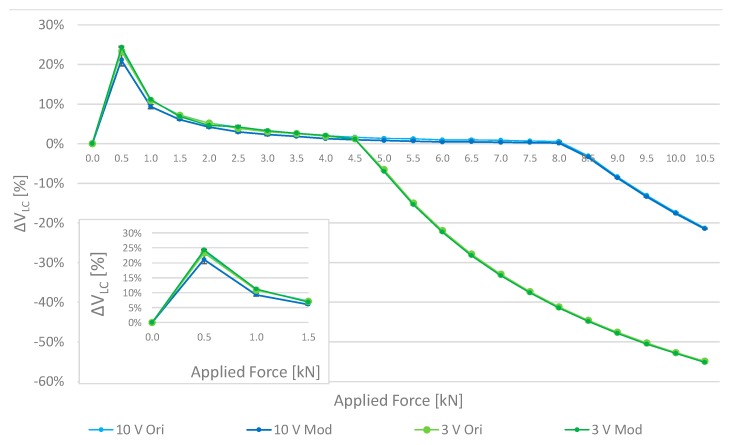
Load cell measured voltage by different applied forces and supplies. “*Ori*” is the LC sensor in a bare configuration, “*Mod*” is the cased one. “*10 V*” and “*3 V*” represent the different sensor supplies.

**Figure 8:**
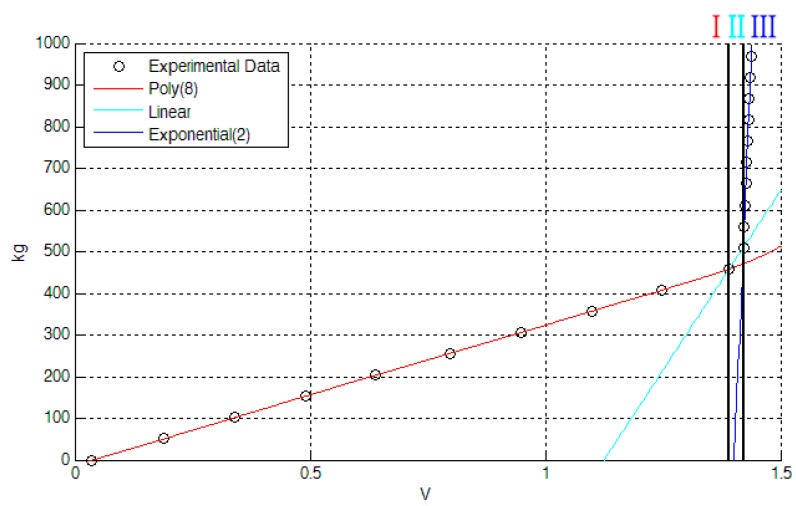
LC calibration curve in the low-power configuration showing the three region curve.
